# Effects of a Multidisciplinary Educational Rehabilitative Intervention in Breast Cancer Survivors: The Role of Body Image on Quality of Life Outcomes

**DOI:** 10.1155/2014/451935

**Published:** 2014-10-28

**Authors:** Giovanni Morone, Marco Iosa, Augusto Fusco, Antonella Scappaticci, Maria Rosaria Alcuri, Vincenzo Maria Saraceni, Stefano Paolucci, Teresa Paolucci

**Affiliations:** ^1^Clinical Laboratory of Experimental Neurorehabilitation, IRCCS Santa Lucia Foundation, Via Ardeatina 306, 00179 Rome, Italy; ^2^Physical Medicine and Rehabilitation, Sapienza University of Rome, Piazzale Aldo Moro 5, 00185 Rome, Italy; ^3^Physical Medicine and Rehabilitation, Policlinico Umberto I, Sapienza University of Rome, Piazzale Aldo Moro 5, 00185 Rome, Italy

## Abstract

In breast cancer survivors, own body image may change due to physical and psychological reasons, worsening women's living. The aim of the study was to investigate whether body image may affect the functional and quality of life outcomes after a multidisciplinary and educational rehabilitative intervention in sixty women with primary nonmetastatic breast cancer who have undergone conservative surgery. To assess the quality of life was administered The European Organization for Research and Treatment of Cancer Study Group on Quality of Life core questionnaire, while to investigate the psychological features and self-image were administered the following scales: the Body Image Scale, the Hamilton Rating Scale for Depression, and the State-Trait Anxiety Inventory. To assess the recovery of the function of the shoulder were administered: the Disabilities of the Arm, Shoulder, and Hand Questionnaire and the Constant-Murley Score. Data were collected at the baseline, at the end of the intervention, and at 3-month follow-up. We found a general improvement in the outcomes related to quality of life, and physical and psychological features after treatment (*P* < 0.001). During follow-up period, a higher further improvement in women without alterations in body image in respect of those with an altered self-perception of their own body was found (*P* = 0.01). In conclusion, the body image may influence the efficacy of a rehabilitative intervention, especially in the short term of follow-up.

## 1. Introduction

Breast cancer (BC) is the most common cancer among women, affecting more than 1.2 million individuals per year worldwide [1]. In recent years, there was a progressive decrement in mortality due to the continuous improvements in early detection of disease and its clinical care [2]. The consequent increasing survival rate implies the growing need for rehabilitative treatments of the short- and long-term sequelae, especially in the upper limb [3]. In the literature, a wide variation in the prevalence of these sequelae has been reported for shoulder pain (from 12 to 51%), for reduction of the range of shoulder motion (from 2 to 51%), and for muscle strength (from 17 to 33%) [4]. All these features may influence the patients' quality of life (QoL) [4], which has been found worsened one year after diagnosis of BC, and that could be influenced in turn by psychological factors, such as body image and mood disturbance [5].

In recent years, a number of growing studies have been focused on body image issues in cancer, such as among BC patients [6]. Body image is an emerging topic in the scientific literature that describes a cognitive dynamic perception of physical appearance of own body [7]. In patients having undergone BC surgery, body image was found altered not only after surgical treatments (mastectomy or partial mastectomy), but also after radiation and/or chemotherapy especially for redness and/or soreness and hair loss and/or weight gain [8]. By now, it should be weighed by surgeons and clinicians in the option of treatment [9, 10]. Low body image, attractiveness, and femininity positively have been found correlated with depression, impacting negatively QoL among women with breast cancer [11].

At the same time, rehabilitation has become an integrative part of the clinical care of people after BC surgery, both for the treatments of motor and for health-related QoL aspects [12]. In fact, exercise interventions are fundamental to improve the motor and psychological sequelae during treatment and in the post treatment rehabilitation period, as recently highlighted by recent Cochrane reviews on this topic [12–14]. A multidisciplinary educational treatment was found to be effective in improving functional abilities and wellbeing and also during the follow-up period after BC surgery [15]. An educational intervention is defined as implying a brief contact with healthcare professionals, self-management patient-led groups, provision of educational booklets, and also Internet discussion groups [16]. It has been shown as psychoeducational group intervention, and physical activity can diminish fatigue and improve energy level, emotional distress at 3-month follow-up, and, finally, the overall QoL following intervention [17].

The direct effect of body image on the effectiveness of a rehabilitative intervention has received far too little attention. It is conceivable that an altered body image may deteriorate the quality of life and functional outcomes of a rehabilitative treatment [18]. Women after BC may experience body shame and being more vulnerable to psychological distress and may experience poorer adjustment after BC treatment [19].

The aim of this study was to investigate the influence of body image on the quality of life related outcomes after a multidisciplinary and educational rehabilitative intervention and at 3-month follow-up in group after breast cancer surgery. At the same time, the secondary aim of our study was to investigate whether the psychological features (as body image, depression, and anxiety) may influence the response to the rehabilitation treatment with objectives like the reduction of pain and the improvement of shoulder function of the operated side.

## 2. Materials and Methods

In the present study we have hypothesized that the alteration of the body image perception could have a negative impact on quality of life and on rehabilitative effectiveness in women affected by the sequelae of breast cancer.

### 2.1. Participants

We have screened consecutive patients referred to physiatrist outpatient consultant for rehabilitation after breast cancer intervention. Inclusion criteria were women with diagnosis of primary nonmetastatic breast cancer having undergone conservative surgery (complete local excision and axillary dissection) or modified radical mastectomy, with age ranging between 18 and 75 years old, with mild to moderate lymphoedema and/or functional limitation of the ipsilateral shoulder. Women were involved in the study at least six months after surgery, and during this period they did not perform any rehabilitative treatment.

Exclusion criteria were active, locoregional recurrence and/or bilateral breast cancer, women without axillary lymph node dissection, concurrent chemotherapy or radiotherapy, preexisting neuromuscular or musculoskeletal conditions affecting local upper extremity testing and/or performance, presence of mastitis and/or lymphangitis, systemic disease, and no previous treatment or contraindication to physiotherapy.

Each patient was assessed at baseline (*T*_0_), at the end of treatment (*T*_end_), and at a three-month follow-up (*T*_f-up_) by the same physician. Efficacy of rehabilitative intervention has been analysed on the entire sample, as detailed below. Then, following the aim of our study, participants were also divided into two groups during data analysis, those with an altered self-perception of body image (Body Image Scale BIS > 10; see below for details) and those without alterations in self-perception of body image (BIS ≤ 10). Among psychological features, anxiety and depression were investigated and analysed.

This trial was approved by the local ethical committee and it was submitted to National Ministry of Health as clinical trial of our institution. An informed consent was signed by patients before participation in the study.

### 2.2. Rehabilitation

Rehabilitation consisted of a multidisciplinary intervention of ten sessions of physiotherapy which lasted around 45 minutes, carried out 2 times a week, into 40 days. Exercise interventions could be defined as moderate to vigorous, in accordance with what was reported by Mishra and colleagues [12]. Sessions were performed by trained physiotherapists on subgroups of patients including 4 or 5 participants each.

Interaction among patients and personal care was encouraged to recreate self-help groups for greater sharing of the experience of illness, and self-management programs could help to increase patients' self-efficacy for better self-management. Physiotherapists tailored rehabilitation regarding patients' functional problems (i.e., lymphedema, reduction of the shoulder range of motion, and pain) and guided the motor rehabilitation. The exercises' protocol is analytically outlined in Table 1. The educational intervention was adapted to enhance the patients' knowledge of the sequelae of BC and to improve the self-care skills to be autonomous in the management of sequelae of BC.

Contents and details were merged with Green's Precede model of health behavior, already used in these patients [20, 21]. The protocol was developed to respond to the collective postoperative care and patients' needs and no personal psychological treatment was planned. Precede model was incorporated into our rehabilitative program by including the use of effective strategies to correct motor habit, promote restorative activities, reduce fatigue, improve patients' knowledge and skills to enable them to perform self-care, promote a balance between activity, exercise, and rest, and finally share with other members of the group beliefs, attitudes, and perceptions.

During the first session, general anatomical details as well as information about lymphedema, limitation of range of motion of the shoulder, and medical and psychological suggestions were provided. Influencing factors, as beliefs, attitudes, and perceptions, were outlined and the following themes were focused on: “what may happen after a breast cancer operation,” “how to prevent lymphedema,” “how to prevent menopausal side effects of hormonal therapy,” “normal crisis reactions,” and “body image and sexual issues.” At the end of the rehabilitation, patients were invited to continue the exercises at home, with the same frequency and in the same way.

### 2.3. Quality of Life Assessment

#### 2.3.1. European Organization for Research and Treatment of Cancer Study Group on Quality of Life Core Questionnaire

The European Organisation for Research and Treatment of Cancer Quality of Life core questionnaire (EORTC QLQ-C30) [22] is the global cancer-specific questionnaire which is used to examine the health-related quality of life among patients with cancer. This is a 30-item core questionnaire, used to assess the physical and psychosocial functioning and symptom experiences. This scale has been validated and revised several times. The questionnaire is divided into five functional scales (physical, role, emotional, cognitive, and social functioning) and nine symptoms' scales and single items (fatigue, nausea/vomiting, pain, dyspnoea, insomnia, appetite loss, constipation, diarrhoea, and financial difficulties). The acquired scores of each scale are spread in the 0–100 domain. A higher score in the functional scales indicates a better function, and in the symptom scales, it indicates a more intensive symptom [23]. For the Italian population, the reliability and the validity were found adequate [24]. The EORTC QLQ-C30 was the primary outcome measure of this study.

### 2.4. Psychometric Assessment

#### 2.4.1. Body Image Scale

The Body Image Scale (BIS) [9] is a 10-item reliable measure developed to assess affective, behavioural, and cognitive dimensions of body image in cancer patients. It uses a 4-point response scale and the final score is the sum of the 10 items, ranging from 0 to 30, with zero scores representing no symptom or distress and higher scores corresponding to increasing symptoms and distress or more body image concerns. We used the median value of the recorded scores to dichotomize them during the analysis of variance. BIS-score was used to dichotomize women in those with altered (BIS-score > 10) and those with unaltered body image (BIS ≤ 10). This division was performed to assess the effect of body image on the effectiveness of therapy in terms of EORTC QLQ-C30 improvement.

#### 2.4.2. Hamilton Rating Scale for Depression

The Hamilton Rating Scale for Depression (HAM-D) [25] is the most popular depression assessment instrument, for both children and adults. It has been demonstrated to be a reliable and a valid standard for symptom burden presence. This scale includes core features of depression, such as low mood and loss of interest in usual activities, and associated features such as anxiety and hypochondriac concerns. At least twenty published versions of the scale exist, including both longer and shortened versions. We used the original version proposed by Hamilton with 17 items, which is the most consistent in detecting change and is validated in Italian [26]. Values higher than 7 were reported as indicating presence of depression [27]; for this reason we have dichotomized the Hamilton scale-scores in 0 if <7 and 1 otherwise for analysis of variance.

#### 2.4.3. State-Trait Anxiety Inventory

The State-Trait Anxiety Inventory (STAI) consists of 20 items referring to self-reported state anxiety and 20 items to trait anxiety [28] and was validated in Italian [29]. It is a reliable and well-validated measure of acute anxiety. The evaluated qualities are feelings of apprehension, tension, nervousness, and worry. Higher scores indicate the presence of anxiety: if the score is more than 40, the trait of anxiety is pathologic [28]. We used this cut-off value to dichotomize the STAI-score during analyses of variance.

### 2.5. Functional Assessment

#### 2.5.1. Disabilities of the Arm, Shoulder, and Hand Questionnaire

The Disabilities of Arm, Shoulder, and Hand Questionnaire (DASH) [30] is a 30-item, self-report questionnaire designed to measure physical functions and symptoms in people with any of several musculoskeletal disorders of the upper limb. It quantifies the general disabilities related to the upper extremity. The items are related to the degree of difficulty in performing various functional activities because of arm, shoulder, or hand troubles (21 items), the severity of pain, activity-related pain, tingling, weakness, and stiffness (5 items), and the effect on social activities, work, and sleep and its psychological impact (4 items). Each item has 5 response options, ranging from 1 to 5. The responses to the 30 items are summed to form a raw score that is then converted to a 0-to-100 scale. A higher score reflects greater disability. The use of Disabilities of Arm, Shoulder, and Hand Questionnaire has rapidly increased in clinical trials and the instrument is available in several languages, Italian included [31].

#### 2.5.2. Constant-Murley Score

The Constant-Murley Score (CMS) is one of the most used, valid, and reliable outcome measures for the assessment of the shoulder [26]. This scoring system consists of subjective variables, such as pain (15 points), activities in daily living (10 points), and arm positioning (10 points) and objective variables, such as range of motion (40 points) and strength (25 points).

#### 2.5.3. Statistical Analysis

**Figure 1:**
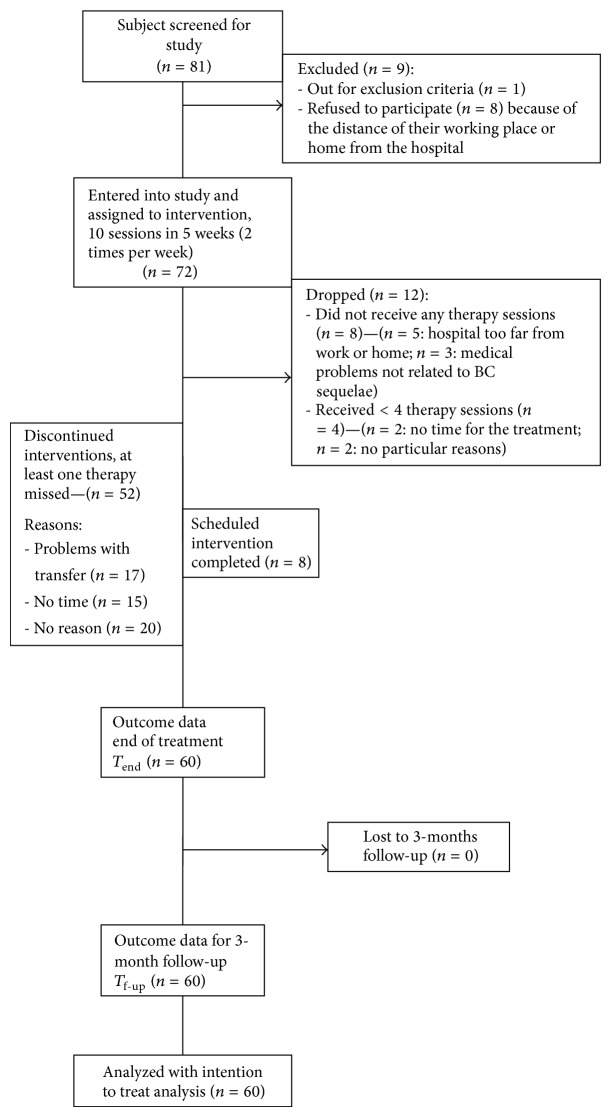
Study flowchart.

An intention to treat analysis was performed on participants who performed at least 4 therapy sessions, as shown in Figure 1. Friedman analyses and Wilcoxon tests for post hoc analyses were performed to assess the variations of scale-scores during time. Mann-Whitney *U* test was performed to test scale-scores between different groups of participants (chemotherapy versus no chemotherapy and mastectomy versus quadrantectomy). Spearman coefficient was computed to assess the correlation between scale-scores. Pearson coefficient (*R*) was computed to assess correlation with the effectiveness, a continuous variable.

An analysis of variance was performed on the European Organisation for Research and Treatment of Cancer QLQ-C30 effectiveness using as factors the three dichotomized psychological scale-scores (Hamilton Rating Scale for Depression >7, State-Trait Anxiety Inventory >40, and Body Image Scale >10). Effectiveness reflects the proportion of potential improvement that was achieved during rehabilitation, calculated as [(discharge score − initial score)/(maximum score − initial score)] × 100. Thus, if a patient achieved the highest possible score after rehabilitation, the effectiveness was 100%.

## 3. Results

### 3.1. Sample Characteristics

During a period of 24 months, eighty-one patients were assessed. Seventy-two patients were enrolled into the study and assigned to the intervention. During treatment, twelve patients were dropped out. None complained about pain. Because these 52 patients did not participate just in a number of sessions between one to a maximum of three, none of them was considered a drop-out, and, applying an intention to treat approach, all the sample of 60 included patients was analysed (see Figure 1). Sample size was not pre-defined, but determined by the period of observation of the study, that is, 2 years.

The mean age of participants was 59.48 ± 11.10 years old. The mean time from previous surgery was 2.1 ± 1.7 years: 45% of the sample have undergone a mastectomy, and 55% have undergone a quadrantectomy. In 72% of the cases, patients have undergone a complete axillary dissection, while the sentinel lymph node has been removed in 27% of them and some lymph node intervention occurred in 1% of the patients. All patients performed a previous period of radiotherapy, while only 55% of the sample performed chemotherapy. Demographic and clinical characteristics are shown in Table 2, including the scales-scores recorded at *T*_0_, at *T*_end_, and at *T*_f-up_.

### 3.2. Changes in Quality of Life and Functional and Psychological Scales

**Figure 2:**
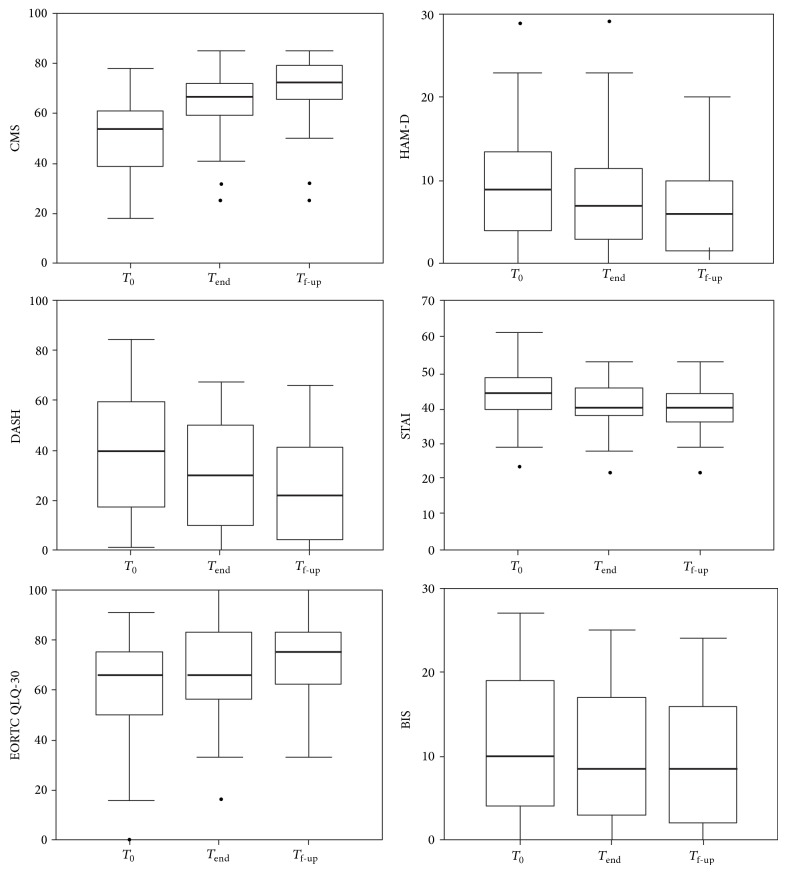
Box and whiskers plot of the scale-scores related to the physical and psychological features of the patients recorded at baseline (*T*_0_), at end of treatment (*T*_end_), and after three months of follow-up (*T*_f-up_). Each box represents first and third quartile values together with the bold line representing the median values; whiskers represent the minimum and maximum values, and the circles are the values considerable by outliers (not excluded but analysed). CMS: Constant-Murley Score, DASH: Disabilities of the Arm, Shoulder, and Hand Questionnaire, EORTC QLQ 30: EORTC Quality of Life core questionnaire, HAM-D: Hamilton Rating Scale for Depression questionnaire, STAI: State-Trait Anxiety Inventory, and BIS: Body Image Scale.

Quality of life of our patients significantly improved after rehabilitation (*χ* ^2^ = 52.29, *P* < 0.001), as shown in Figure 2. This improvement significantly occurred both between *T*_0_ and *T*_end_ (*P* = 0.001) and between *T*_end_ and *T*_f-up_ (*P* = 0.004). The functional scores recorded during the rehabilitative pathway were significantly improved in terms of both CMS (*χ* ^2^ = 103.38, *P* < 0.001, Friedman analysis) and DASH (*χ* ^2^ = 93.27, *P* < 0.001). Post hoc analysis revealed significant improvements observed between *T*_0_ and *T*_end_ for all the scales (*P* < 0.001), and during the follow-up period, a successive improvement was found significant for CMS (*P* < 0.001) and for DASH (*P* < 0.001) (see Figure 2).

The psychological scores were significantly reduced in terms of HAM-D (*χ* ^2^ = 64.50, *P* < 0.001), STAI (*χ* ^2^ = 25.06, *P* < 0.001), and BIS (*χ* ^2^ = 37.26, *P* < 0.001). In particular, for all the recorded scales, a significant improvement in psychological status was observed between *T*_0_ and *T*_end_ (*P* < 0.001 for all of them). During the follow-up period, a successive improvement was found significant for CMS (*P* < 0.001), DASH (*P* < 0.001), EORTC QLQ-C30 (*P* = 0.004), and HAM-D (*P* < 0.001), but neither for BIS- (*P* = 0.043, not statistically significant for Bonferroni correction) nor for STAI-scores (*P* = 0.062). As shown in Table 2 and Figure 2, median values of BIS-score did not change between *T*_end_ and *T*_f-up_, despite quartile values and, hence, the data distributions around medians were quite different in the two assessments.

### 3.3. Factors Affecting Psychological Characteristics at Baseline

At *T*_0_ many correlations were found between psychological scale-scores (BIS and HAM-D: *ρ* = 0.397, *P* = 0.002; BIS and STAI: *ρ* = 0.293, *P* = 0.022, HAM-D; and STAI: *ρ* = 0.565, *P* < 0.001). Only the BIS-score was significantly different between women who underwent a chemotherapy intervention (17; quartiles: 9; 22 versus 5; quartiles: 2; 9,  *P* < 0.001, Mann-Whitney *U* test) and between those who underwent a mastectomy (19; quartiles: 10; 22) versus quadrantectomy (6; quartiles: 3; 11, *P* < 0.001). The STAI-scores (*P* = 0.247, *P* = 0.087, resp.), such as HAM-D scores (*P* = 0.238, *P* = 0.402, resp.), were not affected by these factors. Then, the BIS-score was not affected by the presence of lymphedema (*P* = 0.382).

### 3.4. Effects of Psychological Characteristics on the Quality of Life Related Outcome

**Figure 3:**
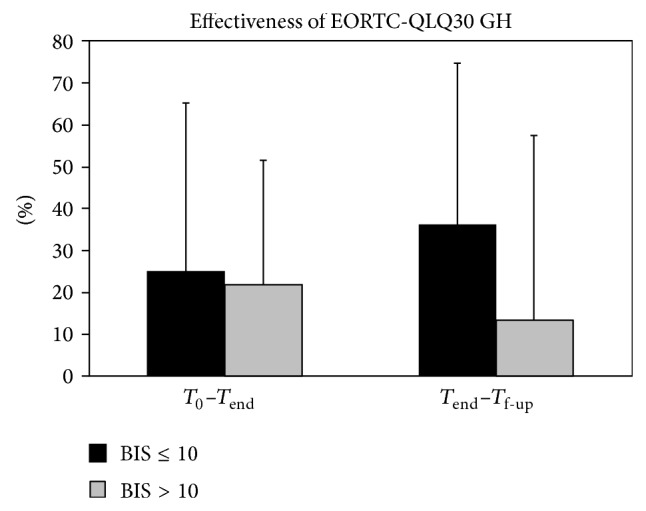
Mean and standard deviation of the effectiveness of the treatment in terms of EORTC QLQ 30 Global Health: EORTC Quality of Life core questionnaire in the treatment period (on the left) and during the follow-up period (on the right) for the patients with Body Image Scale (BIS) scores ≤ 10 (black bars) and >10 (grey bars) at baseline.

A significant correlation with time from surgery was found only for the effectiveness of treatment in terms of STAI (*R* = −0.346, *P* = 0.007): longer was the time from surgery and lower was the effect of treatment on anxiety reduction. An analysis of variance performed on the effectiveness of treatment in terms of EORTC QLQ-C30 general health using BIS-score as binary factor (BIS ≤ 10: not altered body image versus BIS > 10: altered body image) showed a significant effect on the increment during follow-up (i.e., between *T*_end_ and *T*_f-up_) of the BIS-score achieved at the end of treatment *T*_end_  (*F*(1,58) = 5.841, *P* = 0.019), whereas during treatment period (i.e., between *T*_0_ and *T*_end_), the baseline BIS-score (i.e., at *T*_0_) was not significant (*F*(1,58) = 0.124, *P* = 0.726). As shown in Figure 3, the effectiveness of treatment was positive (improvement) between *T*_0_ and *T*_end_ (on the left) and between *T*_end_ and *T*_f-up_ (on the right). If during training all participants, independently from their BIS-score, improved their QoL of about 20% of the maximum possible improvement, in the three months after rehabilitation those with a normal perception of their body image (BIS ≤ 10) seemed to benefit more from the treatment with an ulterior improvement of 30% in their QoL, whereas those with an altered perception of body image had a slight improvement of 10%.

## 4. Discussion

The primary aim of this study was to investigate if the alteration in the body image perception may affect the outcomes related to quality of life after a multidisciplinary educational rehabilitative intervention in women who have undergone breast cancer surgery. We found that, during rehabilitation, body image did not affect the rehabilitative outcomes related to quality of life. Conversely, it had a significant role at follow-up, with a higher further improvement in women without alterations in body image in respect of those with an altered self-perception of their own body (as shown in Figure 3). As expected, both functional and psychological features of the patients improved after rehabilitation. Also the BIS changed significantly during the rehabilitative period, but no further significant changes were recorded during follow-up period. In fact, the effectiveness of treatment was higher for patients with a less altered body image at the follow-up. Although neither body image nor changes in body image were found significantly correlated with time from surgery, this time was found significantly correlated with the benefits obtained in terms of anxiety reduction after treatment, suggesting the need of an early rehabilitative intervention. Body image was found the only factor significantly correlated with the effectiveness of the treatment in terms of quality of life. Neither depression nor anxiety was related to QoL-related outcomes, although before treatment all psychological aspects resulting were strictly related to each other. In particular, body image was found to be influenced by anxiety and depression at the beginning of this study.

Time from surgery was found inversely correlated to the benefits in terms of anxiety reduction after treatment, suggesting the need of an early rehabilitative intervention. Body image was the only factor influenced by the type of surgery (mastectomy or quadrantectomy) and chemotherapy intervention (present or not). Before rehabilitation, our results showed higher levels of alterations in body image, probably related to appearance dissatisfaction and reduced attractiveness, in women treated with mastectomy rather than with quadrantectomy and in those who underwent chemotherapy. In our data, the baseline body image was not influenced by the presence of lymphedema. This result agreed with Speck and colleagues [32] but not with a recent review on this topic [33]. This was probably due to the fact that our patients had a mild stage of lymphedema and were enrolled in mean more than one year after surgery. In add, Speck and colleagues [32] found an improvement in body image perception after rehabilitative intervention (twice a week for 13 weeks), but they did not find any improvement in QoL. Our results suggest that it was the level of body image perception that could affect the further improvement of QoL during the follow-up period. It should be noted that, at the end of the rehabilitation, our patients were invited to continue the exercises at home, with the same cadence and in the same way. But in this period, the lack of a rehabilitative supervision could have increased the affecting role of self-perception.

Our results support the idea that, before the intervention, the medical staff should carefully take into account the body image that each patient experiences. In fact, patients affected by breast cancer have a multidimensionality of the problems like functional, psychological, and social ones which make this disease one of the best candidates for multidisciplinary intervention. The multidimensionality of the problem implied that the intervention changed the body image that, in turn, could affect the effectiveness of the intervention in a complex dance of circular causation. Then, the multidisciplinarity implies the need of an educational part of the intervention as time spent in knowing patients' needs and in providing correct information for managing sequelae and side effects during rehabilitation as well as after rehabilitation with a pamphlet, similarly to previous interventions [34–36]. As strongly affirmed in the cancer rehabilitation, the intervention should aim at achieving full functional, psychological, social, and educational improvement for the patients within the limits imposed by the disease [37]. These psychological features seem to play a fundamental role especially when patients finish their rehabilitative course and they feel more alone and vulnerable.

The findings of this study must be taken into account in light of the limitations. First of all, this study lacks a control group performing another type of intervention. In fact, even if rehabilitation in small groups has been demonstrated to be effective in the motor outcomes for other diseases, as low back pain and osteoporosis [34, 36, 38], it is conceivable that, performing exercises tailored for a single patient, both functional and psychological outcomes could be superior. Then, the interaction among patients and between patients and personal care, which is possible in the small groups, was encouraged but not documented. Hence, this fundamental aspect of the group rehabilitation cannot be precisely analyzed. Finally, the psychological aspects have been taken into account in relation to the body image. Nevertheless, these features may affect the functional outcomes in a wide spectrum of pathologies, from cancer to orthopaedic and neurological diseases [36, 39, 40]. Further studies should better analyze these aspects.

Our sample size of 60 patients is consistent with previous studies present in the literature. At the same time, it has to be noted as it was heterogeneous in terms of time from intervention. This is an aspect quite common for studies in the field of cancer rehabilitation, which could be impacting the results. Further investigations with a broader number of participants involved in these studies, with the use of an appropriate control group and stricter inclusion/exclusion criteria are warranted, especially in this type of patients, who may be evaluated as chronic. For the specific above-cited limits, we can consider our study as a feasibility trial, which highlights the relationship between different aspects of patient experiences. Due to our results, in the future, larger scale randomized controlled trials are needed. In view of the follow-up of just three months, our patients can be considered chronic and the results from the point of view of rehabilitation can be considered stable after these periods of observation. Finally, we did not analyze the effect of age. In fact, recently, it has been demonstrated that even there are significant improvements in physical functioning after a rehabilitative intervention, older women perceived relatively little improvement in QoL compared to younger women, and age had a differential negative influence on improvements in QoL [41]. However, this condition overstates our aim. In the next studies, also demographic and social features (as age, ethnicity, level of education, occupation, marital status, and occupation) should be taken into account as a factor influencing the functionality and QoL-related outcomes after the rehabilitation. Patients work on their body and with their body through specific exercise. Rehabilitation buy a new awareness of the injured part to improve body image.

Nevertheless, our study showed that rehabilitation, even when not performed immediately after the surgical treatment, is effective at the end of the intervention. At follow-up, further improvements were found only in subjects without alterations in body image. Further studies should also plan a psychological treatment during rehabilitative intervention targeted to improve body image, which has been shown by our results to be a prognostic factor for quality of life related outcomes.

## 5. Conclusions

Our results showed that the body image perception did not affect the efficacy of a rehabilitative intervention from baseline to the end of treatment, but it affected the potential further improvement occurring during the follow-up period, when women were probably more vulnerable. Then, we found that body image, depression, and anxiety resulting are significantly correlated to each other at baseline. Despite this, body image was the only factor affected by the type of surgical intervention and chemotherapy and it was the only factor affecting quality of life related outcomes.

In conclusion, considering the psychological aspects of the patients before planning a patient-tailored rehabilitative pathway may improve the efficacy of the rehabilitative intervention. In particular, own body image perception should be considered another treatment target for maintaining the improved level of quality of life of these patients.

## Figures and Tables

**Table 1:** Exercises' program.

Exercise	Description
Exercise 1 Relaxing	Diaphragmatic breathing and postural elements such as alignment of the midline. The patient is supine on a mat with flexed knees and arms along the chest.

Exercise 2 Raising arm, opening and closing punches	Lying on the bed, arms at sides, flexed knees. Slowly, lift parallel the arms till the upright position, opening and clenching the fists in order to contract the muscles of the arms. Maintain up the position, opening and closing the punches five times, and return to the starting position. Repeat ten times.

Exercise 3 Stretching and releasing the arms. Recovery of the flexion.	Lying on the bed, flexed knees. Interlock the fingers and slowly bring to full flexion and then on the pillow; then put as much as possible while keeping your arms extended. Perform the technique of breathing for a minute. Repeat ten times.

Exercise 4 Turn the shoulders. Rotation-anteropulsion-retropulsion	Standing or sitting, rotate the shoulders for ten times.

Exercise 5 Abduction and adduction of the arms—isometric strengthening	Lying or standing, bring the arms out, with extended elbows. Maintain the position for six seconds. Return to the starting position, slowly. Repeat for ten times.

Exercise 6 Opening and closing elbows	Standing, legs apart. Place both hands on the hips with thumbs pointing backwards. Push your elbows back and forth without moving the hands. Repeat for ten times.

Exercise 7 Run up the wall—recovery of flexion	Getting closer to the maximum height possible completely to the wall, stopping for few seconds, and trying to gently remove the hands from the wall. Repeat ten times.

Exercise 8 Run up the wall by the side—recovery of abduction	With a bit of tape, standing with the unaffected side facing the wall, place the hand on the wall at shoulder height. Reaching a point with his hand, as high as possible, marking it with the piece of tape. Begin the exercise with the operated arm, and repeat ten times trying to get closer to the mark left on the wall.

Exercise 9 Hand on the operated shoulder—recovery of the adduction	Sitting or standing, arms along the sides. Bring the hand of the operated limb to the contralateral shoulder. Return to starting position. Repeat ten times.

Exercise 10 Rotate the arms—promoting the rotations	Standing with legs apart, raise your arms to the shoulder. Beginning with small movements, gradually increase the diameter up to do it without complaints. We recommend at least five full rotations. Finally, bring the arm down the side and remain at rest for a moment.

Exercise 11 Run up the back—promoting the extension and intrarotation	Standing, place the hands behind the back and take with the healthy hand, the hand of the operated limb. Slowly slide the hands along the spine upwards to its possible and maintain the position for a few seconds. Then decline slowly. Repeat for ten times.

Exercise 12 Opening and closing the elbows	Standing or sitting, with the feet firmly on the ground, interlock the hands ahead of face with the head upright. Slowly, raise the arms above the head and then behind the neck. Then open both elbows and close them for five times. If you feel discomfort at the wound, keep the position and work with the breathing.

Exercise 13 Bar	Sitting or standing, hold the bar (about 110 cm long). Bring the arms forward and upward at the elbows extended. Flex elbows and bring the bar behind the head. Return slowly to starting position. Repeat ten times.

Exercise 14 Codman's pendulum	Lying on a bed in a prone position, the arm perpendicular to the floor, make a circular motion clockwise and counterclockwise slowly. Repeat for ten times.

Exercise 15 Flex on the front wall—to prevent the deficit of the scapula	Standing in front of the wall, put the hands against the wall parallel. Flex the arms. Repeat for ten times.

**Table 2:** Demographic and clinical characteristics of the sample. Cases (and percentages) or mean ± standard deviation of demographic and clinical characteristics of the sample. Median (1st; 3rd quartiles) for the scale scores. *T*_0_: baseline; *T*_end_: end of treatment; *T*_f-up_: three-month follow-up; QoL: quality of life; DASH: Disabilities of the Arm, Shoulder, and Hand Questionnaire; BIS: Body Image Scale; HAM-D: Hamilton Rating Scale for Depression; CMS: Constant-Murley Score; EORTC QLQ-C30: Quality of Life core questionnaire (GH: global health; FS: function scale; SS: symptom scale); STAI: State-Trait Anxiety Inventory.

Demographic characteristics			
Number of patients		60	
Age [years]		59.48 ± 11.10	
Body mass index [kg/m^2^]		26.42 ± 3.85	
Married/common-law wife		46 (77%)	
Working, not employed, or retired		43 (72%)	
High school or master degree education		36 (60%)	
Clinical characteristics			
Quadrantectomy		33 (55%)	
Mastectomy		27 (45%)	
Chemotherapy		33 (55%)	
Radiotherapy		60 (100%)	
Lymphatic drainage		10 (17%)	
Years from surgery [years]		2.11 ± 1.70	
Scale-scores	*T*_0_	*T*_end_	*T*_f-up_
EORTC QLQ-30 GH	66 (50; 75)	66 (57; 83)	75 (64; 83)
EORTC QLQ-30 FS	76 (58; 83)	83 (72; 87)	84 (71; 92)
EORTC QLQ-30 SS	20 (13, 28)	13 (7; 21)	11 (3; 18)
CMS	54 (39; 61)	67 (60; 72)	73 (66; 79)
DASH	40 (17; 59)	30 (10; 48)	18 (4; 40)
HAM-D	9 (4; 11)	7 (3; 11)	6 (1; 10)
STAI	44 (40; 48)	40 (38; 45)	40 (36; 44)
BIS	10 (4; 19)	8 (3; 17)	8 (2; 16)
Effectiveness	*T*_0_–*T*_end_		*T*_end_–*T*_f-up_
EORTC QLQ-30 GH	16 (0; 32)		15 (0; 32)
EORTC QLQ-30 FS	15 (7; 35)		9 (0; 24)
EORTC QLQ-30 SS	−7 (−11; −3)		−2 (−6; 0)
